# Comparative Study of the Gut Microbiota Among Four Different Marine Mammals in an Aquarium

**DOI:** 10.3389/fmicb.2021.769012

**Published:** 2021-10-21

**Authors:** Shijie Bai, Peijun Zhang, Changhao Zhang, Jiang Du, Xinyi Du, Chengwei Zhu, Jun Liu, Peiyu Xie, Songhai Li

**Affiliations:** ^1^Institute of Deep-sea Science and Engineering, Chinese Academy of Sciences, Sanya, China; ^2^Marine Mammal and Marine Bioacoustics Laboratory, Institute of Deep-sea Science and Engineering, Chinese Academy of Sciences, Sanya, China; ^3^Atlantis, Sanya, China; ^4^Key Laboratory of Tropical Translational Medicine of Ministry of Education, Hainan Medical University, Haikou, China; ^5^University of Chinese Academy of Sciences, Beijing, China

**Keywords:** gut microbial communities, beluga, Pacific white-sided dolphin, common bottlenose dolphin, Cape fur seal

## Abstract

Despite an increasing appreciation in the importance of host–microbe interactions in ecological and evolutionary processes, information on the gut microbial communities of some marine mammals is still lacking. Moreover, whether diet, environment, or host phylogeny has the greatest impact on microbial community structure is still unknown. To fill part of this knowledge gap, we exploited a natural experiment provided by an aquarium with belugas (*Delphinapterus leucas*) affiliated with family Monodontidae, Pacific white-sided dolphins (*Lagenorhynchus obliquidens*) and common bottlenose dolphin (*Tursiops truncatus*) affiliated with family Delphinidae, and Cape fur seals (*Arctocephalus pusillus pusillus*) affiliated with family Otariidae. Results show significant differences in microbial community composition of whales, dolphins, and fur seals and indicate that host phylogeny (family level) plays the most important role in shaping the microbial communities, rather than food and environment. In general, the gut microbial communities of dolphins had significantly lower diversity compared to that of whales and fur seals. Overall, the gut microbial communities were mainly composed of Firmicutes and Gammaproteobacteria, together with some from Bacteroidetes, Fusobacteria, and Epsilonbacteraeota. However, specific bacterial lineages were differentially distributed among the marine mammal groups. For instance, *Lachnospiraceae*, *Ruminococcaceae*, and *Peptostreptococcaceae* were the dominant bacterial lineages in the gut of belugas, while for Cape fur seals, *Moraxellaceae* and *Bacteroidaceae* were the main bacterial lineages. Moreover, gut microbial communities in both Pacific white-sided dolphins and common bottlenose dolphins were dominated by a number of pathogenic bacteria, including *Clostridium perfringens*, *Vibrio fluvialis*, and *Morganella morganii*, reflecting the poor health condition of these animals. Although there is a growing recognition of the role microorganisms play in the gut of marine mammals, current knowledge about these microbial communities is still severely lacking. Large-scale research studies should be undertaken to reveal the roles played by the gut microbiota of different marine mammal species.

## Introduction

Microorganisms, as a resident group that has colonized the mammalian body, especially the gut, outnumber mammalian cells by as many as ten to one, and therefore encode 100-fold more unique genes than the genome of their hosts (Ley et al., 2006). The gut microbiota and their hosts always evolve together, forming a symbiotic immune system, where the microbiota plays an essential role in the development stage of the immune system, functioning both locally and systemically (Dang and Marsland, 2019). For instance, host immune systems have complex mechanisms to exclude invading pathogens that interact with the hosts. Furthermore, many physiological processes, such as obesity, energy metabolism, blood pressure, glucose homeostasis, clotting risks, and different behaviors of hosts, are influenced by gut microbiota. The metabolites generated by gut microbes and host’s receptors work together through a crucial cross talk between the gut microbiota and different organs to response their different phenotypes (Schroeder and Bäckhed, 2016). The majority of microbes residing in the gut have a profound influence on host physiology and nutrition, both of which are crucial for host health (Bäckhed et al., 2005; Flint et al., 2012). Therefore, revealing the gut microbiota of mammals is vital to fully understand the physiological and health status of mammals themselves. To date, the majority of research has focused on the gut microbiota of humans with very limited information on the composition of gut microbiota from other mammals, especially marine mammals, often due to sampling constraints.

Belugas (*Delphinapterus leucas*), affiliated with family Monodontidae, are omnivorous toothed cetaceans, and the most abundant odontoceti in Arctic waters. The Arctic cod (*Boreogadus saida*) is the most important food source of some beluga populations, such as the Beaufort Sea beluga population (Loseto et al., 2009). However, redfish (*Sebastes marinus*), halibut (*Reinhardtius hippoglossoides*), shrimp (*Pandalus borealis*), saffron cod (*Eleginus gracilis*), rainbow smelt (*Osmerus mordax*) and Pacific salmon (*Oncorhynchus* spp.), are also considered as the potential food of belugas (Frost and Lowry, 1981; Heide-Jorgensen and Teilmann, 1994; Quakenbush et al., 2015). The Pacific white-sided dolphin (*Lagenorhynchus obliquidens*) and the common bottlenose dolphin (*Tursiops truncatus*) are delphinids. The Pacific white-sided dolphins are one of the most abundant, widely distributed small delphinids in cold temperate waters of the North Pacific Ocean, and their scope of activities includes the eastern North Pacific, ranging from the Gulf of California to the Gulf of Alaska, and the western North Pacific, including the East China Sea, Sea of Japan, and Sea of Okhotsk (Hayano et al., 2004). Pacific white-sided dolphins primarily consume high-energy fishes, including anchovy, sardine, herring, and salmon, and other prey such as squid (Rechsteiner et al., 2013). The common bottlenose dolphins are globally distributed in warm temperate waters as one of the top predators in the marine food chain (Wells and Scott, 1999). Their diet composition includes different fish, such as the herring and mackerel (*Scomber scombrus*), and other organisms like squid (Kastelein et al., 2002). The Cape fur seals (*Arctocephalus pusillus pusillus*), affiliated with familly Otariidae (Kirkman and Arnould, 2018), are the only pinniped endemic to the African continent and widely distribute in the range from the southeast coast of South Africa to southern Angola (Kirkman et al., 2013). Their food source includes teleost fish, cephalopods, elasmobranchs, and seabirds, specifically including hake (*Merluccius* spp.), sardine (*Sardinops sagax*), chokka squid (*Loligo vulgaris reynaudii*), horse mackerel (*Trachurus trachurus capensis*), anchovy (*Engraulis encrasicolus*), and West Coast rock lobster (*Jasuslalandii*) (David, 1987; Makhado et al., 2006; Mecenero et al., 2006). Phylogeny of these three marine mammal lineages is shown in Supplementary Figure S1 (modified from Yuan et al., 2021).

Food is not only essential for the survival and growth of mammals but also impacts the symbiotic microbial communities in the gut. Food can shape the gut microbiota, modulating microbial composition and function, impact host–microbe interactions, and lead to changes in health status (McKenzie et al., 2017). Environment is another important factor, which impacts gut microbiota diversity, as underlying environmental changes will either increase or decrease the diversity of gut microbiota (McKenzie et al., 2017; Makki et al., 2018). A network-based analysis of microbial communities from the fecal microbiota of humans and 59 other mammalian species indicated that both host diet and phylogeny impact microbial diversity and composition, the intensity of influence increasing from carnivory to omnivory to herbivory. However, no marine mammals were included in that study (Ley et al., 2008). A subsequent study found that the gut microbiomes of different mammalian lineages have diverged at equally rate over the past 75 million years, but the gut microbiomes of Cetartiodactyla (ruminants, whales, and hippopotami) have evolved much faster than that of Chiropterans, and the gut microbiota are more likely to be associated with a strict mammalian lineage than with a particular dietary category, resulting in a strong phylogenetic influence on gut microbiomes (Nishida and Ochman, 2018). So far, research focused on the gut microbiota of marine mammals has primarily been based on next generation sequencing technologies, rather than the culture-dependent techniques, the latter of which just can reveal only a small fraction of total microbial communities of target samples (Bai et al., 2021; Glaeser et al., 2021). To the best of our knowledge, there have been no studies regarding the gut microbiota of Belugas, Pacific white-sided dolphins, and Cape fur seals. However, few studies have explored the gut microbial communities of common bottlenose dolphins, including captive and wild individuals, based on culture-independent approaches, which have shown that the Proteobacteria (Gamma), Firmicutes, and Fusobacteria dominate (Suzuki et al., 2019; Robles-Malagamba et al., 2020).

The first step toward developing health indices for the rapid assessment of marine mammal health is to explore their gut microbial communities, especially for those housed in aquariums, which are sampled on a regular basis. Characterizing the microbial communities of different marine mammals in aquariums can provide insight into their health status. The aim of this study was to explore the gut microbiota of four different marine mammal species in a shared environment with the same food sources, including herring (*Clupea harengus*) and capelin (*Mallotus villosus*). Thirty-five fecal samples from two whales, four dolphins, and nine fur seals were used in this study to address two questions: (i) what are the gut microbial compositions and diversity of belugas, Pacific white-sided dolphins, and Cape fur seals; moreover, what are the similarities and differences of gut microbial community structure and composition of common bottlenose dolphins collected from the aquarium in Sanya, China, with previous studies? and (ii) with the same living environment and food sources, could we find the similar gut microbial communities between different marine mammal species? Of food sources, environment, and phylogeny, which is the most influential factor that shapes the gut microbiota?

## Materials and Methods

### Sample Collection

Two belugas, three Pacific white-sided dolphins, one common bottlenose dolphin, and nine Cape fur seals kept in the Marine and Waterpark in Atlantis hotel, Sanya, China, were surveyed in this study (Supplementary Table S1). All animals were maintained for public display in indoor pools with natural seawater filtered by the same filtration system based on mechanical filtration with percolator filters, pressurized sand filter, and protein skimmers. All seawater was treated with chlorination and ozone sterilization. The diet of all surveyed animals consisted of whole frozen fish including herring (*Clupea harengus*) and capelin (*Mallotus villosus*).

For each individual animal, at least one fecal sample was collected during June to July, 2020. All 35 fecal samples were collected from animals under operant conditioning (behavioral training) during routine medical examinations. Fecal samples were harvested by veterinarians between 8:00 and 9:00 in the morning. Whales and dolphins were asked for a “belly-up” position and feces were collected directly from the rectum with a Levin’s tube (Vygon, France), with a diameter of 4mm, inserted 10–20cm into the rectum. Fecal samples of fur seals were collected using anal swabs, with a diameter of 12mm, and inserted 10–15cm into the rectum. All fecal samples were frozen at −20°C until analysis.

### DNA Extraction and Sequencing

DNA was extracted using MoBio PowerSoil extraction kits (Mo Bio Laboratories, Carlsbad, CA, United States), according to the manufacturer’s instructions. Three extraction blank control samples were also included. The extracted DNA was quantified with a Qubit fluorometer (Invitrogen Inc. Manufacturer: Life Technologies Holdings Pte Ltd., Singapore) and used for amplification of the V4 region of the 16S rRNA gene with the primer pair 515f Modified and 806r Modified (Walters et al., 2015). The PCR cycling conditions were as follows: denaturation at 95°C for 3min, followed by 27cycles at 95°C for 30s, 55°C for 30s, and 72°C for 45s, and a final extension at 72°C for 10min. Triplicate PCR amplifications were combined after purification with a TaKaRa purification kit (TaKaRa, Japan). The PCR products were prepared for library construction with the TruSeq DNA sample preparation kit (Illumina, San Diego, CA, United States), according to the manufacturer’s instructions. The libraries were sequenced at MajorBio Co. Ltd. (Shanghai, China) using the HiSeq platform (Illumina) with a paired-end 250bp sequence read run.

### Microbial Community Analysis

After sequencing, the raw reads were assigned to their respective samples according to their barcodes and forward and reverse primers (one mismatch of each was allowed). Paired-end reads of sufficient length with at least a 30-bp overlap were combined into full-length sequences by using FLASH program version 1.2.8 (Magoč and Salzberg, 2011). The average fragment length was 253bp. Btrim program (version 0.2.0) was used to filter out low-quality sequences. The quality score was set to >20 with a 5-base window size as the standard and any sequences containing Ns or<200bp were discarded. Sequences with lengths of 245–260bp were retained (Kong, 2011). We used UNOISE3 to correct sequencing errors to determine the real biological sequences at single-nucleotide resolution by generating amplicon sequence variants (ASVs) with default settings (Edgar, 2016). A representative sequence from each ASV was selected for taxonomic annotation by comparison with the SILVA 132 database (Quast et al., 2013), which includes bacterial, archaeal, and eukaryotic sequences. To account for varying sequencing depths, the ASVs were randomly resampled to normalize the reads of each sample. The raw sequencing reads of all samples were deposited to the NCBI database^1^ under BioProject accession number: PRJNA743584.

## Statistical Analysis

The diversity of the microbial communities from the fecal samples of different marine mamamals were determined by statistical analysis of the α-diversity indices. The Shannon and Inverse Simpson indices were calculated using the vegan package in R language version 3.4.3 (R Core Team, 2018). The Chao1 values (Chao, 1984) were generated using the Mothur program (Schloss et al., 2009). The indicator ASVs for microbial communities were classified using IndVal.g analysis with the R package labdsv tool (Roberts, 2007). Only ASVs with highly significant indicator values (IndVal.g index >0.95, *p*<0.001) were considered as strict habitat specialists (Li et al., 2021). To investigate differences in microbial composition among fecal samples, ß-diversity-based statistical tools and non-metric multidimensional scaling (NMDS) were used for calculating Bray-Curtis and Jaccard distance matrices. We also tested whether there were any dissimilarities among defined groupings, including belugas, Pacific white-sided dolphins, common bottlenose dolphin, and Cape fur seals, by performing permutational multivariate analysis of variance (PERMANOVA), multi response permutation procedure (MRPP), and a one-way ordered analysis of similarity (ANOSIM). Data analyses followed the method previously described in Bai and Hou (Bai and Hou, 2020). Data comparison between different groups was performed by the Wilcoxon rank-sum test using IBM SPSS Statistics 19.

## Results

### Sequencing Statistics and Microbial Diversity

**Figure 1:**
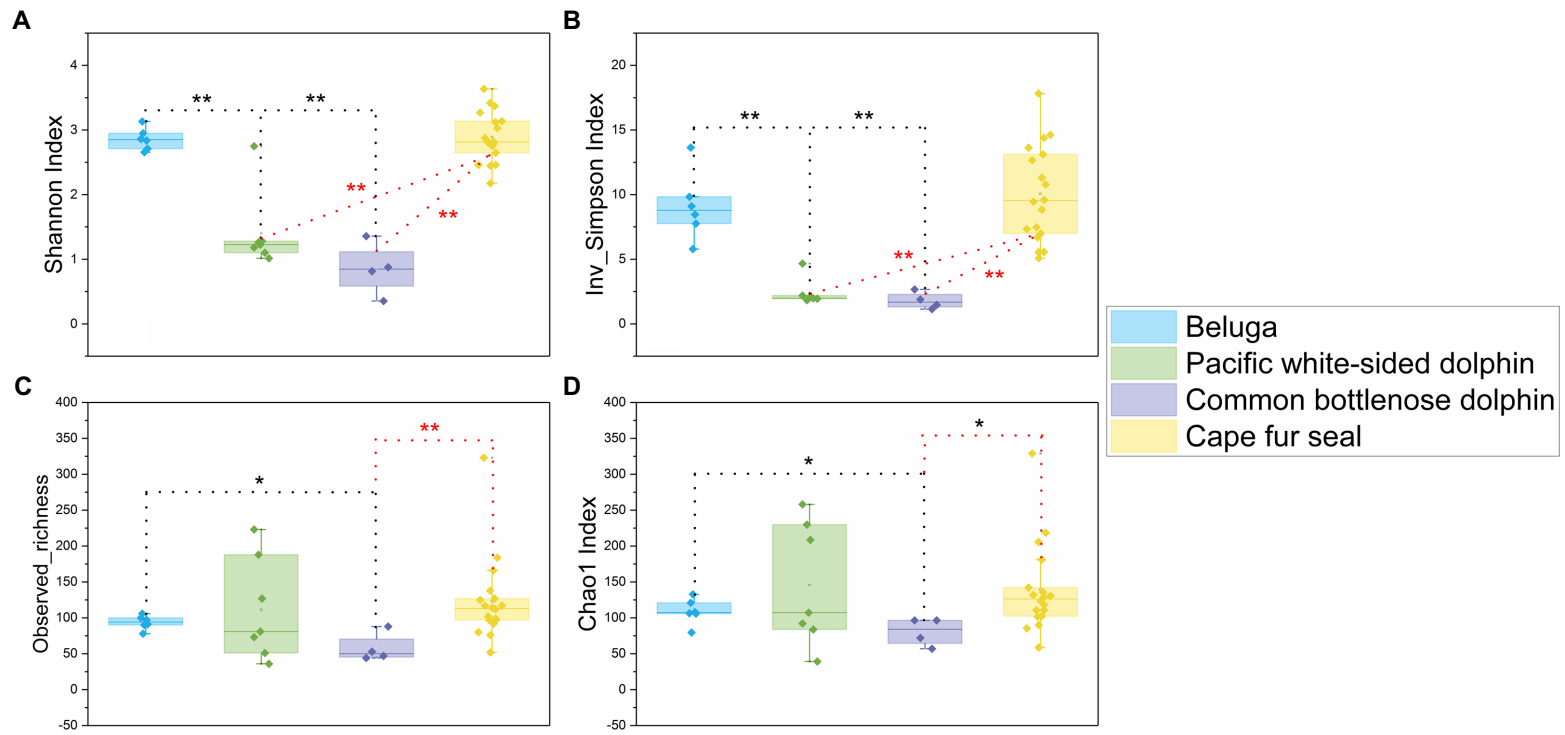
Comparisons of four α-diversity indices, Shannon index **(A)**, Inverse Simpson index **(B)**, observed richness **(C)**, and Chao1 index **(D)**, of the 35 fecal specimens from belugas, Pacific white-sided dolphins, common bottlenose dolphin, and Cape fur seals. Based on Wilcoxon rank-sum test, *difference is significant at 0.05 level, **difference is significant at 0.01 level. The results are based on the ASVs datasets.

A total of 1,927,942 sequences were obtained from 35 fecal samples of different marine mammals after quality assessment, the marine mammals included two belugas, three Pacific white-sided dolphins, one common bottlenose dolphin, and nine Cape fur seals. An average of 55,084±10,935 sequences was obtained from each sample. In order to obtain a more accurate result of α-diversity, we rarefied to 33,334 sequences per sample, and then used this set for analysis of microbial diversity, composition, and structure. The α-diversities of microbial communities from the gut of different marine mammals were calculated. The Shannon, Inverse Simpson, and Chao1 indices and observed richness indicated that the α-diversity of the gut microbiomes from belugas and Cape fur seals was higher than those from Pacific white-sided dolphins and common bottlenose dolphin (Figure 1).

### Structure and Composition of the Microbial Communities

**Figure 2:**
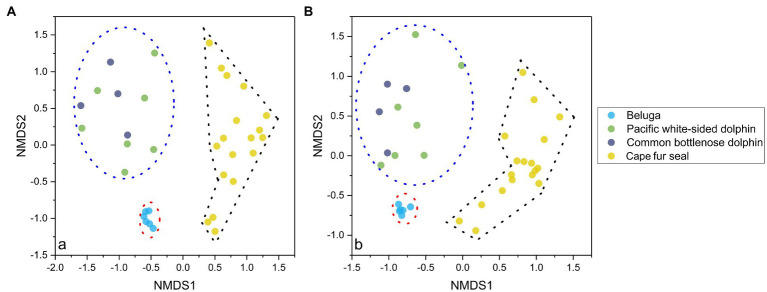
NMDS analysis of the gut microbial communities separated the samples into three principal groups, the first group composed of the fecal samples of belugas, the second group composed of fecal samples from the Pacific white-sided dolphins and common bottlenose dolphin, and the third group composed of fecal samples from Cape fur seals. The results are based on the ASVs datasets, and the left **(A)** and right **(B)** plots were calculated based on Bray-Curtis distance and Jaccard distance, respectively.

NMDS analysis of microbial communities clearly showed three principal groups: belugas, two dolphin species, and Cape fur seals (Figure 2). These results suggest that whales, dolphins, and fur seals possess different gut microbial communities, even in the context of similar food and environment. Furthermore, the MRPP, ANOSIM, and PERMANOVA showed significant differences in the gut microbial communities of whales, dolphins, and fur seals (*p*<0.01). By contrast, no significant difference was detected between the Pacific white-sided dolphin and the common bottlenose dolphin (*p*>0.05; Table 1).

**Table 1:** Dissimilarity tests of gut microbial communities of whales, dolphins, and fur seals based on Bray-Curtis and Jaccard distance.

Group	Bray-Curtis	Jaccard
	*p*	*p*
**Group (Whales and Dolphins)**
	PERMANOVA	0.001(^*^), *F*=7.569	0.001(^*^), *F*=6.056
MRPP	0.001(^*^), R=0.636	0.001(^*^), R=0.583
ANOSIM	0.001(^*^), R=0.577	0.001(^*^), R=0.326
**Group (Whales and Fur seals)**
	PERMANOVA	0.001(^*^), *F*=16.03	0.001(^*^), *F*=12.71
MRPP	0.001(^*^), R=0.559	0.001(^*^), R=0.557
ANOSIM	0.001(^*^), R=0.829	0.001(^*^), R=0.827
**Group (Dolphins and Fur seals)**
	PERMANOVA	0.001(^*^), *F*=10.93	0.001(^*^), *F*=8.715
MRPP	0.001(^*^), R=0.705	0.001(^*^), R=0.685
ANOSIM	0.001(^*^), R=0.919	0.001(^*^), R=0.872
**Group (Pacific white-sided dolphins and common bottlenose dolphin)**
	PERMANOVA	0.085, *F*=1.988	0.061, *F*=1.454
MRPP	0.090, R=0.758	0.062, R=0.727
ANOSIM	0.078, R=0.259	0.158, R=0.135

* *Difference is significant at 0.001 level. Whales refer to belugas; dolphins refer to Pacific white-sided dolphins and common bottlenose dolphin; and fur seals refer to Cape fur seals*.

**Figure 3:**
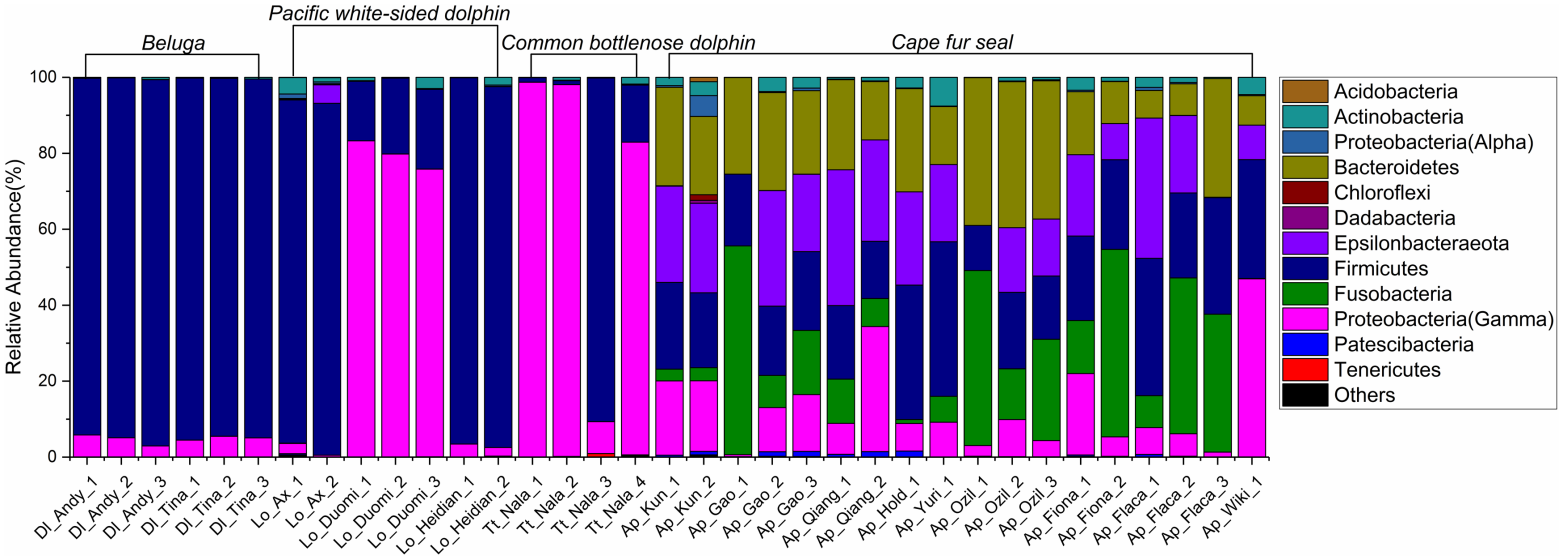
Gut microbial community members of belugas, Pacific white-sided dolphins, common bottlenose dolphin, and Cape fur seals at the phylum level.

**Figure 4:**
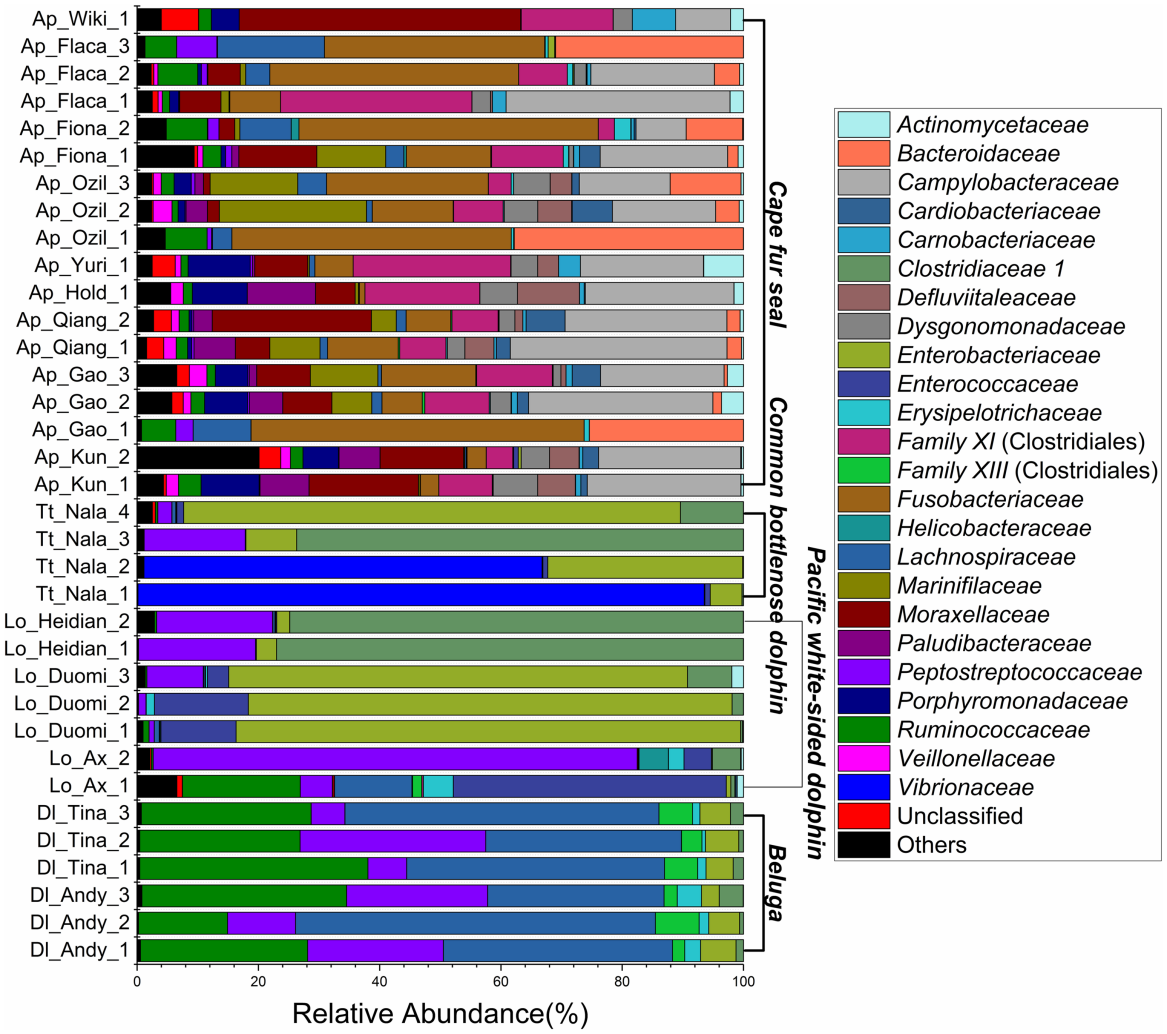
Gut microbial community members of belugas, Pacific white-sided dolphins, common bottlenose dolphin, and Cape fur seals at the family level.

**Figure 5:**
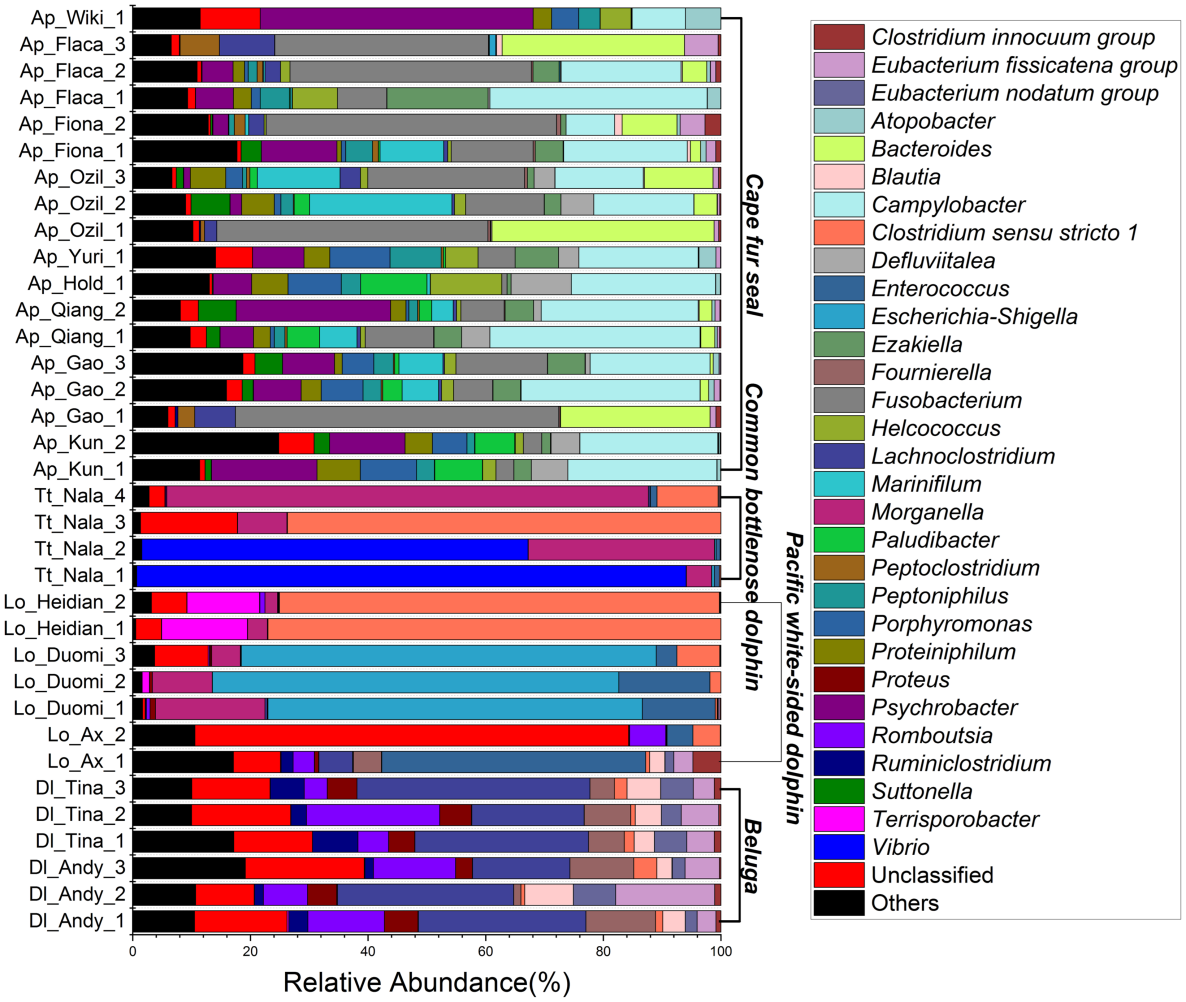
Gut microbial community members of belugas, Pacific white-sided dolphins, common bottlenose dolphin, and Cape fur seals at the genus level.

The relative abundance of microorganisms was evident at the phylum, family, and genus levels with a similarity of 97% for ASV classification and provided detailed information on the composition of the microbial communities (Figures 3–5). Firmicutes were the dominant bacterial lineage in the fecal samples of belugas, accounting for 94%~96%. The dominant microbial phyla for two of the three Pacific white-sided dolphins were also Firmicutes, the remaining animal (Duomi) was dominated by Proteobacteria (Gamma) (76%~83%). The majority of the fecal samples from the lone common bottlenose dolphin in this study were dominated by Proteobacteria (Gamma), with one exception that was dominated by Firmicutes. The gut microbial communities of Cape fur seals were dominated by Firmicutes, Bacteroidetes, Fusobacteria, Epsilonbacteraeota, and Proteobacteria (Gamma). However, the respective compositions of different seal fecal samples were slightly different. At the family taxonomic level, *Lachnospiraceae*, *Ruminococcaceae*, and *Peptostreptococcaceae*, which are affiliated with the Clostridiales, were the dominant bacterial lineages in the beluga fecal samples. *Enterobacteriaceae*, *Enterococcaceae*, *Clostridiaceae 1*, and *Peptostreptococcaceae* were dominant in the fecal samples of Pacific white-sided dolphins. The gut microbial communities of the common bottlenose dolphin was also dominated by *Enterobacteriaceae*, *Clostridiaceae 1*, and *Peptostreptococcaceae*, but *Vibrionaceae* was also one of the dominant bacterial lineage in some of the fecal samples. *Campylobacteraceae*, *Fusobacteriaceae*, *Family XI* (affiliated with the Clostridiales), *Moraxellaceae*, and *Bacteroidaceae* were the dominant bacterial lineages in the fecal samples of Cape fur seals. Furthermore, at the genus taxonomic level, the gut microbial communities of belugas were mainly composed of *Lachnoclostridium*, *Romboutsia*, *Fournierella*, and *Eubacterium fissicatena* group. Meanwhile, *Escherichia-Shigella*, *Clostridium sensu stricto 1*, and *Enterococcus* were the dominant bacterial genera in the fecal samples of Pacific white-sided dolphins, while *Vibrio*, *Morganella*, and *Clostridium sensu stricto 1* were dominant in samples from the common bottlenose dolphin. As for Cape fur seals, *Fusobacterium*, *Campylobacter*, *Psychrobacter*, *Bacteroides*, *Marinifilum*, and *Ezakiella* were the main bacterial genera.

### Gut Microbial Indicators of Different Marine Mammals

**Figure 6:**
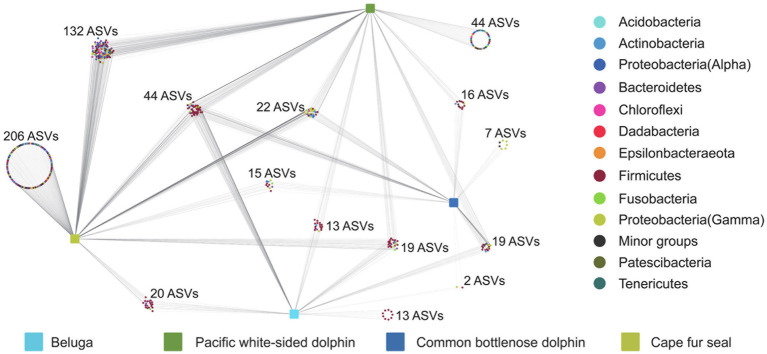
Distribution of ASVs in the gut of belugas, Pacific white-sided dolphins, common bottlenose dolphin, and Cape fur seals.

To better understand the core microbial communities of different marine mammals, we explored the detailed differences at the ASV level between different marine mammal fecal samples. Quality control and random resampling of the 35 samples were conducted, and the sequence reads were clustered into 572 ASVs at the 97% similarity level. As shown in Figure 6, in the fecal samples of belugas, Pacific white-sided dolphins, common bottlenose dolphin, and Cape fur seals, contained 13, 44, 7, and 206 unique ASVs, respectively. Cape fur seals shared 132, 20, and 15 ASVs with Pacific white-sided dolphins, belugas, and common bottlenose dolphin, respectively, while Pacific white-sided dolphins shared 16 and 13 ASVs with common bottlenose dolphin and belugas, respectively. By contrast, only two ASVs were shared between belugas and common bottlenose dolphin. Furthermore, 44 ASVs were detected in all four different marine mammal species.

**Figure 7:**
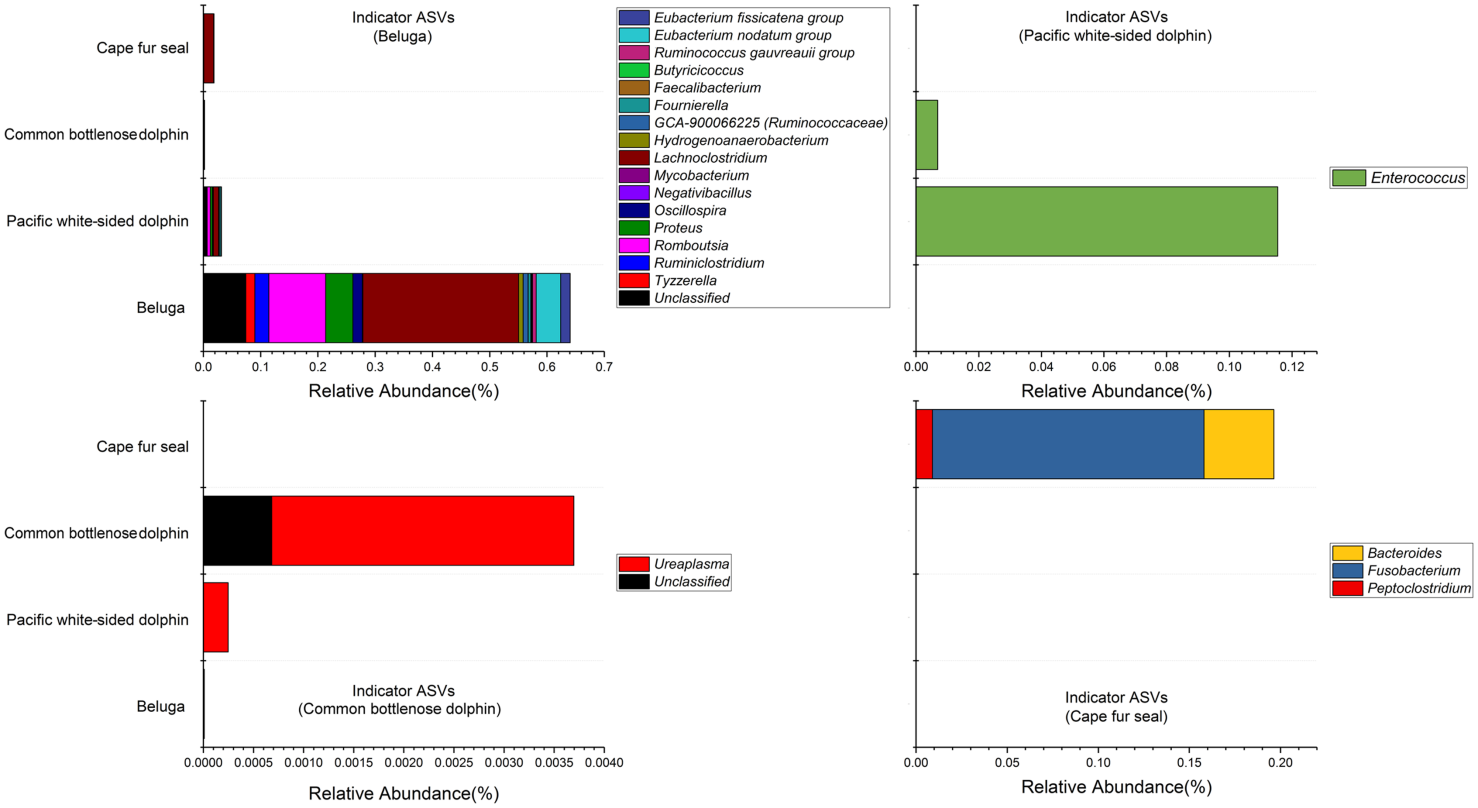
Stacked bar chart showing relative abundance of indicator ASVs in the gut of belugas, Pacific white-sided dolphins, common bottlenose dolphin, and Cape fur seals. The indicator organisms being shown was at the genus level.

**Figure 8:**
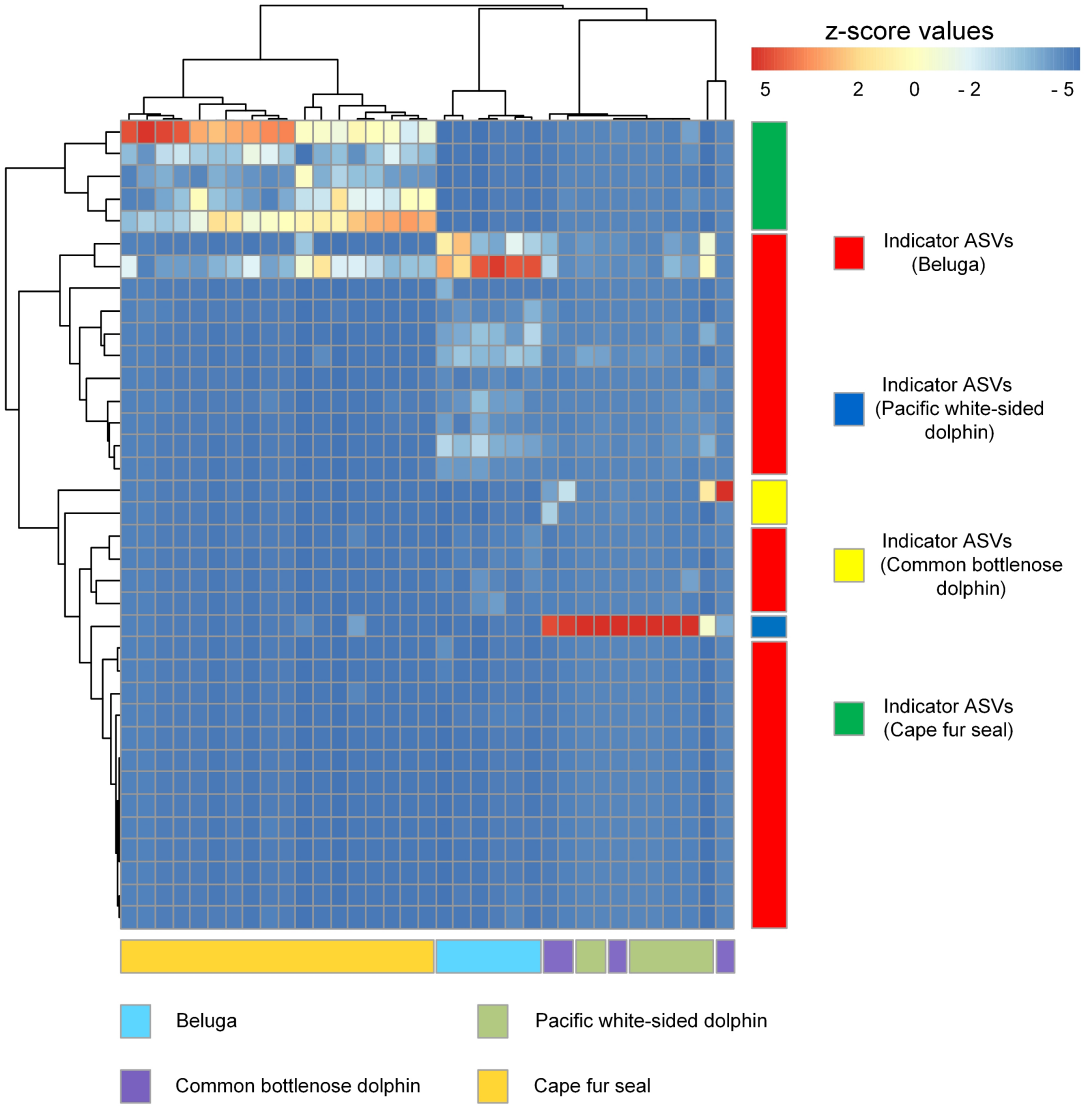
Heatmap diagram showing the distribution of indicator ASVs of belugas, Pacific white-sided dolphins, common bottlenose dolphin, and Cape fur seals. Each row and column of the heatmap diagram corresponds to a single indicator and samples, respectively. The row data for each indicator were z-score transformed. Dendrograms were constructed based on Pearson correlation clustering.

Indicator organisms analysis showed that *Lachnoclostridium*, *Proteus*, *Eubacterium nodatum group*, *Tyzzerella*, *Ruminiclostridium*, *Oscillospira*, *Eubacterium fissicatena group*, *Romboutsia*, *Hydrogenoanaerobacterium*, *GCA-900066225* (*Ruminococcaceae*), *Ruminococcus gauvreauii group*, *Negativibacillus*, *Mycobacterium*, *Fournierella*, *Faecalibacterium*, *Butyricicoccus*, and some other unclassified genera were highly associated with the gut microbial communities of belugas (Figure 7). *Enterococcus* was the only indicator genus in the gut microbial communities of Pacific white-sided dolphins. In the gut of the common bottlenose dolphin, *Ureaplasma* and one unclassified genus were the indicator organisms. Three indicator genera, *Fusobacterium*, *Bacteroides*, and *Peptoclostridium*, were observed in the fecal samples of Cape fur seals. The heatmap based on these indicator ASVs reveals that belugas and Cape fur seals harbor distinct gut microbial communities (Figure 8). Meanwhile, the gut microbial communities of Pacific white-sided dolphins and common bottlenose dolphins could not be separated (Figure 8), consistent with the NMDS results.

## Discussion

### Main Factors Affecting the Gut Microbial Community of Different Marine Mammals in the Same Aquarium With Same Food Resources

In this study, we reported the gut microbial communities in two belugas, three Pacific white-sided dolphins, one common bottlenose dolphin, and nine Cape fur seals. These marine mammals resided at the Marine and Waterpark in Atlantis hotel, Sanya, China. Moreover, these creatures also shared the same water filtration system and diet including herring (*Clupea harengus*) and capelin (*Mallotus villosus*). The α-diversity indices showed that the gut microbial diversity of belugas and Cape fur seals were higher than that of Pacific white-sided dolphins and common bottlenose dolphin, with the gut microbial diversity of the two dolphin species being similar. Given the same food sources and water quality, we speculate that the low gut microbial diversity of Pacific white-sided dolphins and common bottlenose dolphin may attribute to the poor health status of the animals themselves.

In their pioneering work, Ley et al. (2008) explored the fecal microbiota of humans and 59 other mammalian species living in captivity and in the wild. Results indicated that host diet and phylogeny both influence microbial diversity, and the influence increases from carnivory to omnivory, and to herbivory (Ley et al., 2008). The gut microbiota makes an important contribution to host metabolism by contributing enzymes that are not encoded by the host genome such as the decomposition of polysaccharides and polyphenols and the synthesis of vitamins (Rowland et al., 2018). Different diets can shape gut microbial communities in both the short and long term (Flint et al., 2012). For instance, low intake of accessible carbohydrates and dietary fibers may reduce the microbial diversity and change the microbial communities, contributing to the depletion of specific microbial taxa (Sonnenburg and Sonnenburg, 2014; Sonnenburg et al., 2016). In our study, four different marine mammals are carnivores, living in the same environment, and eating the same food. Therefore, if diet and environment affects gut microbial community composition and structure more than the phylogeny, the gut microbial β-diversity of belugas, Pacific white-sided dolphins, common bottlenose dolphin, and Cape fur seals should tend to be similar. However, according to the results of NMDS and different statistical analyses, belugas, dolphins, and Cape fur seals possess significantly different gut microbial communities. Whereas, the gut microbial communities of Pacific white-sided dolphins and common bottlenose dolphin were statistically undifferentiated, we propose the reason for the similarities in β-diversity may attributed to the animals’ health status and medical treatments prior to sampling; however, further study is required.

### Microbial Community Composition and Keystone Species in the Gut of Belugas, Pacific White-Sided Dolphins, Common Bottlenose Dolphins, and Cape Fur Seals

Members of Firmicutes, Bacteroidetes, Fusobacteria, and Epsilonbacteraeota constituted the vast majority of the microorganisms in the gut of Cape fur seals. The most common bacterial genera were *Fusobacterium*, *Campylobacter*, *Psychrobacter*, *Bacteroides*, *Marinifilum*, and *Ezakiella*. However, to date, no Cape fur seal gut microbial communities have been reported. The skin microbial communities of Antarctic fur seal (*Arctocephalus gazella*) showed that Proteobacteria, Bacteroidetes, Firmicutes, and Actinobacteria were the dominant bacterial lineages (Grosser et al., 2019). Although there are definite similarities, the Fusobacteria, rather than the Actinobacteria were the dominant bacterial lineage in the gut of Cape fur seals. Nevertheless, at the genus level, only *Psychrobacter* was detected as a dominant bacterial lineage both in the gut and the skin microbial communities of fur seals, and *Chryseobacterium* and *Jeotgalibaca*, which were dominant in the skin microbial communities, were either not detected (*Chryseobacterium*) in the gut microbial dataset or identified as a single ASV (*Jeotgalibaca*) within in the gut of AX (0.003–0.05%, relative abundance, Pacific white-sided dolphin), and Kun, Flaca (0.003–0.02%, relative abundance, Cape fur seals). The *Fusobacterium*, as an animal-associated genus affiliated with the *Fusobacteriaceae*, are widely found in the gut of humans and other animals (Brennan and Garrett, 2019) including rats (Zhang et al., 2020), macaques (Nugeyre et al., 2019), pigs (Wylensek et al., 2020), penguins (Tian et al., 2021), striped dolphin (Godoy-Vitorino et al., 2017), and whales (Li et al., 2019), and some birds and fishes (Michl et al., 2017; Rasheeda et al., 2017; Chen et al., 2018; Michel et al., 2018). These organisms may play a symbiotic or pathogenic role (Brennan and Garrett, 2019).

The genus *Campylobacter* are very common bacteria in animal digestive tracts, whose powerful flagellar motility is very important in nutrient acquisition, whereby bacterial cells attach to surfaces and rotate their flagella to increase nutrient flux for metabolism (Beeby, 2015). Different species of Campylobacter can have variable characteristics of either pathogen or commensal, depending on their host immune status and the pathotype of the bacteria themselves (Wigley, 2015). For example members of the Campylobacter, such as Campylobacter jejuni and Campylobacter coli, are among some of the most common human gastrointestinal pathogens, causing bacterial diarrheal illness (Fitzgerald, 2015). In addition, the ASVs classified into *Campylobacter* in this study were further identified by phylogenetic analysis. The results showed that these ASVs clustered together far from the common species of *Campylobacter* (Supplementary Figure S2). Therefore, the role of *Campylobacter* in the gut of Cape fur seals remains unclear. The members of the *Psychrobacter*, another common genus, can be found in different marine environments and marine creatures, such as Arctic fjord (Zeng et al., 2015), Arctic glacier (Zeng et al., 2016), deep sea (Evans-Yamamoto et al., 2019), penguins (Kämpfer et al., 2020), corals (Zachariah et al., 2016), and marine crustaceans (Romanenko et al., 2009). In this and previous studies, the genus *Psychrobacter* was dominant in the gut of Cape fur seals and the skin of Antarctic fur seal (*Arctocephalus gazella*). At the same time, we barely detected this bacterium in the gut of belugas, Pacific white-sided dolphins, and common bottlenose dolphin. The members of *Psychrobacter*, as one of the commensal bacteria, could improve the microbial diversity of gastrointestinal tract, and upregulate the expression of immune-related genes and is therefore considered to be probiotic (Sun et al., 2011; Yang et al., 2011; Makled et al., 2017). According to previous study, it is interesting that the decrease in the relative abundance of *Psychrobacter* may associate with depressive conditions of hosts. Meanwhile, the results of metabolomics showed that the lower levels of amino acids, and fatty acids, and higher amounts of bile acids, hypoxanthine, and stercobilins were detected in the depression groups. However, the roles of these bacteria in the different stages of depression are not clear (Yu et al., 2017). Moreover, the *Psychrobacter*, one of the key genera of young-like gut microbial community of African turquoise killifish (*Nothobranchius furzeri*), was considered to play an important role in extending host life span (Smith et al., 2017). Additionally, after infection by porcine epidemic diarrhea virus, the relative abundance of *Psychrobacter*, which is a symbiotic group of bacteria in the gut of pigs, was decreased.

Members of the Bacteroidetes, one of the major lineages of friendly bacterial commensuals, possess large numbers of genes encoding carbohydrate active enzymes, which allows them to switch readily between different energy sources in the gut, depending on availability, using sophisticated regulatory mechanisms to control gene expression (Thomas et al., 2011). Polysaccharides comprise the most abundant type of biological polymer and, as such, also the most abundant biological food source. Carbohydrate fermentation by Bacteroides and other intestinal bacteria results in the production of a pool of volatile fatty acids that are absorbed through the large intestine and utilized by the host as an energy source, providing a significant proportion of the host’s daily energy requirement (Hooper et al., 2002). Although most of the Bacteroides are commensals in the gut, some species can also be responsible for infection, including *Bacteroides fragilis*, *Bacteroides distasonis*, *Bacteroides ovatus*, *Bacteroides thetaiotaomicron*, *Bacteroides vulgatus*, and *Bacteroides uniformis*, with significant morbidity and mortality (Wexler, 2007). However, in this study, the genus *Bacteroides* was only found to be dominant in the fecal samples of Cape fur seals and was rarely detected in the other three marine mammals. Moreover, ASV 12, ASV 24, and ASV 35 were the most predominant ASVs and were classified to *Bacteroides* at the genus level. The phylogenetic analysis, based on the neighbor-joining method, showed that the strain *Bacteroides plebeius* DSM 17135 was most closely related to ASV 12, ASV 24, and ASV 35 (Supplementary Figure S3). *Bacteroides plebeius* is a common bacterium in human faces, with numerous strains (Kitahara et al., 2005). Furthermore, compared to healthy cats, the relative abundance of *B*. *plebeius* was significantly decreased in the fecal microbiome of animals with Feline chronic enteropathy (CE; Marsilio et al., 2019). The genus of *Marinifilum* is often isolated or detected in coastal sea water and sediments (Na et al., 2009; Ruvira et al., 2013; Xu et al., 2016) and is most likely involved in the hydrolysis and fermentation of proteins, carbohydrates, and lipids (Müller et al., 2018). The members of *Ezakiella*, a genus of Gram-stain positive, coccus-shaped, anaerobic bacteria are often found in the intestinal and vaginal tracts of healthy people (Patel et al., 2015; Diop et al., 2018). However, based on the very limited reports currently available, it is not clear what role(s) this genus may play in the gut and vagina.

The bacterial genus of *Lachnoclostridium* is commonly found in the healthy human gut (Tidjani Alou et al., 2016a,b; Traore et al., 2017). But, *Lachnoclostridium* was also isolated from the gut microbiota of an obese patient (Amadou et al., 2016). A previous study reported that the genus of *Romboutsia* was predominant in the samples of mammalian gut microbiota (Gerritsen et al., 2018). However, this genus can be treated as one of the gut microbiotal markers for obesity in patients (Zeng et al., 2019). The *Fournierella* can be found in the fecal samples of humans, and it has also been reported to be significantly associated with intrahepatic fat accumulation (Yaskolka Meir et al., 2021). The *Eubacterium fissicatena* group is closely associated with obesity and obesity-related metabolic disorders of the host. Previous studies found that medium-, long-, and medium-chain (MLM) structured lipids have anti-obesity effects (Song et al., 2021). After supplementation with MLM structured lipids, the relative abundance of *E*. *fissicatena* group were decreased (Yue et al., 2020). The dominant gut bacterial lineages of belugas in this study indicated that they were obese or overweight, which we propose to be an essential environmental adaption strategy for the belugas to defense extremely cold in the arctic waters.

*Escherichia-Shigella*, *Clostridium sensu stricto 1*, and *Enterococcus* were the dominant bacterial lineages in the fecal samples of Pacific white-sided dolphins. However, only one ASV (ASV3) was assigned to *Escherichia-Shigella*. The results of phylogenetic analysis indicate that ASV3 is closely related to either *Cronobacter turicensis* or *Erwinia iniecta* (Supplementary Figure S4). *Cronobacter turicensis* is an opportunistic foodborne pathogen, which can lead to severe disease manifestations, such as brain abscesses, meningitis, necrotizing enterocolitis, and systemic sepsis (Lehner and Stephan, 2004; Stephan et al., 2011). Only one paper has reported that *E*. *iniecta* possess virulence to the Russian wheat aphid (Campillo et al., 2015). The most abundant ASV, ASV2, was assigned to *Clostridium sensu stricto 1* at the genus level and was closely related to *Clostridium perfringens* based on the phylogenetic analysis (Supplementary Figure S5). ASV11 was present in the fecal samples of Pacific white-sided dolphins and was closely related to *Enterococcus lactis* (Supplementary Figure S6), which can produce an antimicrobial substance that is stable over a wide range of pH (2–10) and after heating at 100°C for 15min (Ben Braïek et al., 2018a). Therefore, *E*. *lactis* is classified as a probiotic (Sharma et al., 2012; Ben Braïek et al., 2018b). *Clostridium perfringens* is a typical anaerobic, Gram-positive, spore-forming bacillus that can secrete more than 20 virulence toxins and has been associated with intestinal diseases ranging in severity from diarrhea to necrotizing enterocolitis and myonecrosis in both animals and humans (Ohtani and Shimizu, 2016; Kiu and Hall, 2018). *Clostridium perfringens* was found to be dominant in the fecal samples of the common bottlenose dolphin of this study. The other two dominant bacterial lineages were *Vibrio* and *Morganella*. Phylogenetic analysis showed that ASV40 was closely related to *Vibrio fluvialis* (Supplementary Figure S7), and ASV 5 was closely related to *Morganella morganii* (Supplementary Figure S8). *Vibrio fluvialis* and *M*. *morganii* are both pathogenic bacteria. *Vibrio fluvialis* is a halophilic, Gram-negative pathogen commonly found in coastal environments including sea water and seafood. Several outbreaks and sporadic cases of acute diarrhea caused by *V*. *fluvialis* have been reported extensively (Igbinosa and Okoh, 2010; Chowdhury et al., 2012, 2013; Liang et al., 2013). *Morganella morganii* is a gram-negative bacterium found in the gut of humans and is associated with many diseases including cellulitis, abscessation, sepsis, bacteremia, and diarrhea (Falagas et al., 2006; Kim et al., 2007; Liu et al., 2016). These results indicated that the Pacific white-sided dolphins and common bottlenose dolphin are in poor health condition.

Indicator species refers to a species constrained to one or a few habitat types, and as “specialists” have a greater susceptibility to local or regional extinction. These organisms can also potentially represent a better ecological indicator of environmental change than habitat generalists (Carignan and Villard, 2002). For example, in the vegetation studies, the species of plant that are absolutely inclined to present in a single or, at the most, a few vegetation types are identified as indicator species, and this method is beneficial to the identification of vegetation types in research surveys (Chytrý et al., 2002). To find out the association between single species and one or several groups of sites representing habitat types, community types, or other categories, the indicator value index (IndVal) is used to assess these relationships (De Cáceres and Legendre, 2009). Because there were an uneven number of fecal samples from the different groups of marine mammals in this study, we used the index of IndVal.g to eliminate the effect of different numbers in each group. According to the set threshold (IndVal.g>0.95, *p*<0.001), our results show that the gut microbial communities of belugas harbor 28 indicator species representing by ASVs. While five and two ASVs were identified in the gut microbial communities of Cape fur seals and common bottlenose dolphin, respectively. However, only a single ASV was detected in the gut microbiota of Pacific white-sided dolphins. Heatmap results based on these indicator species show that different species of marine mammals can harbor distinct microbial communities with the exception of Pacific white-sided dolphins and common bottlenose dolphin, which cannot be separated. Interestingly, in our study, the microbial phyla with the highest relative abundance in the fecal samples of common bottlenose dolphin were Proteobacteria (Gamma) and Firmicutes at the phylum level, and *Vibrionaceae*, *Enterobacteriaceae*, *Clostridiaceae 1*, and *Peptostreptococcaceae* at the family level. These findings were consistent with previous studies (Soverini et al., 2016;Suzuki et al., 2019 ; Robles-Malagamba et al., 2020). The *Fusobacteriaceae* of Fusobacteria was also documented in the previous studies as a dominant bacterial lineage (Suzuki et al., 2019; Robles-Malagamba et al., 2020), however, we barely detected Fusobacteria in the gut microbial communities of common bottlenose dolphin but found that it dominated the fecal samples of Cape fur seals. Moreover, at the genus level, previous work has shown that the genera *Cetobacterium* (70.9%), *Clostridium XI* (15.3%), and *Clostridium sensu stricto* (4.2%) had the highest relative abundance in the fecal samples of common bottlenose dolphins, while in our microbial datasets, *Vibrio* (*Vibrio fluvialis*), *Morganella (Morganella morganii)*, and *Clostridium sensu stricto 1 (Clostridium perfringens)* were the dominant bacterial lineages, all of which are pathogenic bacteria, especially the *C*. *perfringens*, which have been reported to be the causative agent of common bottlenose dolphins (Buck et al., 1987; Jaing et al., 2015). Furthermore, we also detected lager number of *C*. *perfringens* in the fecal samples of Pacific white-sided dolphins, because the *C*. *perfringens* can enter other dolphins *via* skin wounds, which serve as the focus for these bacteria to penetrate and spread (Buck et al., 1987). Meanwhile, the spread of same bacteria between Pacific white-sided dolphins and common bottlenose dolphin may contribute the similarity of microbial community structure between them. Unfortunately, we did not find any published reports on the gut microbiota of Pacific white-sided dolphins. For a comparative and comprehensive understanding, further studies of the microbial communities of Pacific white-sided dolphins from different places should be conducted in the future.

## Conclusion

In this study, we systematically compared gut microbiota of four different marine mammals in an aquarium; they shared the same food, and depended on the same water filtration system. We found the belugas and Cape fur seals harbored remarkably greater gut microbial diversity than Pacific white-sided dolphins and common bottlenose dolphin. Diet, environment, and host phylogeny, all of these have been reported to have impact on microbial community structure. Our results demonstrated that phylogeny (>family level) has the greatest influence on the shaping of microbial communities. Although diet and water quality were the same for all subjects, we still observed significantly different gut microbial community structure among whales, dolphins, and fur seals. Nevertheless, the microbial communities of Pacific white-sided dolphins and common bottlenose dolphin showed similarities, but most of the dominant bacterial lineages were pathogenic bacteria revealed by phylogenetic analysis. Moreover, some potential pathogens were found in the guts of both Pacific white-sided dolphins and common bottlenose dolphin, and this could be one of the reasons for the similarity in gut microbial community structure of these two dolphins. Our results also indicated that the Pacific white-sided dolphins and common bottlenose dolphin are in poor health condition, and that the gut microbiota of belugas could aid in defense against cold conditions. Our study provides foundamental information on the composition of gut microbial communities in marine mammals; however, knowledge about this subject is still scarce. For better understanding of the gut microbial community structure and composition of marine mammals, further studies should be focused on the spatial and temporal of gut microbiota in different marine mammal species in the future.

## Data Availability Statement

The datasets presented in this study can be found in online repositories. The names of the repository/repositories and accession number(s) can be found at: https://www.ncbi.nlm.nih.gov/, PRJNA743584.

## Ethics Statement

The animal study was performed under an Ethical Statement from Institute of Deep-sea Science and Engineering, CAS, with the number of IDSSE-SYLL-MMMBL-01.

## Author Contributions

SB, PZ, and SL conceived and designed this study. PZ, CZ, XD, JL, and PX collected the samples. SB did the experiments, prepared the figures, and wrote the manuscript. SB, JD, and CZ analyzed the data. PZ and SL revised the manuscript. All authors contributed to the article and approved the submitted version.

## Funding

This project was supported by grants of the Youth Innovation Promotion Association of Chinese Academy of Sciences (2020363).

## Conflict of Interest

CZ and XD are employed by Atlantis Sanya.

The remaining authors declare that the research was conducted in the absence of any commercial or financial relationships that could be construed as a potential conflict of interest.

## Publisher’s Note

All claims expressed in this article are solely those of the authors and do not necessarily represent those of their affiliated organizations, or those of the publisher, the editors and the reviewers. Any product that may be evaluated in this article, or claim that may be made by its manufacturer, is not guaranteed or endorsed by the publisher.

## Footnotes

^1^ http://www.ncbi.nlm.nih.gov/
