# Supplementary material for: Comparative Study of the Gut Microbiota Among Four Different Marine Mammals in an Aquarium
Source: Front Microbiol. 2021 Oct 21;12:769012. doi: 10.3389/fmicb.2021.769012 (PMC8567075; doi:10.3389/fmicb.2021.769012)
Supplement: Supplementary file 1 [file Data_Sheet_1.docx]

**Table S1.** Sampling information of the four different marine mammals during the 2020 from Marine and Waterpark, Atlantis hotel, Sanya, China, including: Sample ID, Sampling data, Species name, Latin name, Sex, Age, Country of origin.

| Individual  ID | Sample ID | Sampling date | Species name | Latin name | Sex | Age | Country of origin |
| --- | --- | --- | --- | --- | --- | --- | --- |
| Andy | Andy_1 | June 25th | Beluga | *Delphinapterus leucas* | M | 6 | Russia |
|  | Andy_2 | July 9th | Beluga | *Delphinapterus leucas* | M | 6 | Russia |
|  | Andy_3 | July 22th | Beluga | *Delphinapterus leucas* | M | 6 | Russia |
| Tina | Tina_1 | June 25th | Beluga | *Delphinapterus leucas* | F | 6 | Russia |
|  | Tina_2 | July 9th | Beluga | *Delphinapterus leucas* | F | 6 | Russia |
|  | Tina_3 | July 22th | Beluga | *Delphinapterus leucas* | F | 6 | Russia |
| AX | AX_1 | June 23th | Pacific white-sided dolphin | *Lagenorhynchus obliquidens* | F | 6 | Japan |
|  | AX_2 | July 9th | Pacific white-sided dolphin | *Lagenorhynchus obliquidens* | F | 6 | Japan |
| Duomi | Duomi_1 | June 23th | Pacific white-sided dolphin | *Lagenorhynchus obliquidens* | F | 6 | Japan |
|  | Duomi_2 | July 9th | Pacific white-sided dolphin | *Lagenorhynchus obliquidens* | F | 6 | Japan |
|  | Duomi_3 | July 22th | Pacific white-sided dolphin | *Lagenorhynchus obliquidens* | F | 6 | Japan |
| Heidian | Heidian_1 | June 23th | Pacific white-sided dolphin | *Lagenorhynchus obliquidens* | F | 6 | Japan |
|  | Heidian_2 | July 9th | Pacific white-sided dolphin | *Lagenorhynchus obliquidens* | F | 6 | Japan |
|  | Heidian_3 | July 22th | Pacific white-sided dolphin | *Lagenorhynchus obliquidens* | F | 6 | Japan |
| Nala | Nala_1 | June 23th | Common bottlenose dolphin | *Tursiops truncatus* | F | 6 | Japan |
|  | Nala_2 | July 9th | Common bottlenose dolphin | *Tursiops truncatus* | F | 6 | Japan |
|  | Nala_3 | July 22th | Common bottlenose dolphin | *Tursiops truncatus* | F | 6 | Japan |
|  | Nala_4 | July 29th | Common bottlenose dolphin | *Tursiops truncatus* | F | 6 | Japan |
| Kun | Kun_1 | July 14th | Cape fur seal | *Arctocephalus pusillus pusillus* | F | 4 | Angola |
|  | Kun_2 | July 22th | Cape fur seal | *Arctocephalus pusillus pusillus* | F | 4 | Angola |
| Gao | Gao_1 | June 24th | Cape fur seal | *Arctocephalus pusillus pusillus* | M | 4 | Angola |
|  | Gao_2 | July 14th | Cape fur seal | *Arctocephalus pusillus pusillus* | M | 4 | Angola |
|  | Gao_3 | July 22th | Cape fur seal | *Arctocephalus pusillus pusillus* | M | 4 | Angola |
| Qiang | Qiang_1 | July 14th | Cape fur seal | *Arctocephalus pusillus pusillus* | M | 4 | Angola |
|  | Qiang_2 | July 22th | Cape fur seal | *Arctocephalus pusillus pusillus* | M | 4 | Angola |
| Ho | Ho_1 | July 14th | Cape fur seal | *Arctocephalus pusillus pusillus* | F | 4 | Angola |
| Yuri | Yuri_1 | July 14th | Cape fur seal | *Arctocephalus pusillus pusillus* | F | 4 | Angola |
| Ozil | Ozil_1 | June 24th | Cape fur seal | *Arctocephalus pusillus pusillus* | M | 4 | Angola |
|  | Ozil_2 | July 14th | Cape fur seal | *Arctocephalus pusillus pusillus* | M | 4 | Angola |
|  | Ozil_3 | July 22th | Cape fur seal | *Arctocephalus pusillus pusillus* | M | 4 | Angola |
| Fiona | Fiona_1 | July 14th | Cape fur seal | *Arctocephalus pusillus pusillus* | M | 4 | Angola |
|  | Fiona_2 | July 22th | Cape fur seal | *Arctocephalus pusillus pusillus* | M | 4 | Angola |
| Flaca | Flaca_1 | June 24th | Cape fur seal | *Arctocephalus pusillus pusillus* | M | 4 | Angola |
|  | Flaca_2 | July 14th | Cape fur seal | *Arctocephalus pusillus pusillus* | M | 4 | Angola |
|  | Flaca_3 | July 22th | Cape fur seal | *Arctocephalus pusillus pusillus* | M | 4 | Angola |
| Wiki | Wiki_1 | July 22th | Cape fur seal | *Arctocephalus pusillus pusillus* | F | 4 | Angola |

*
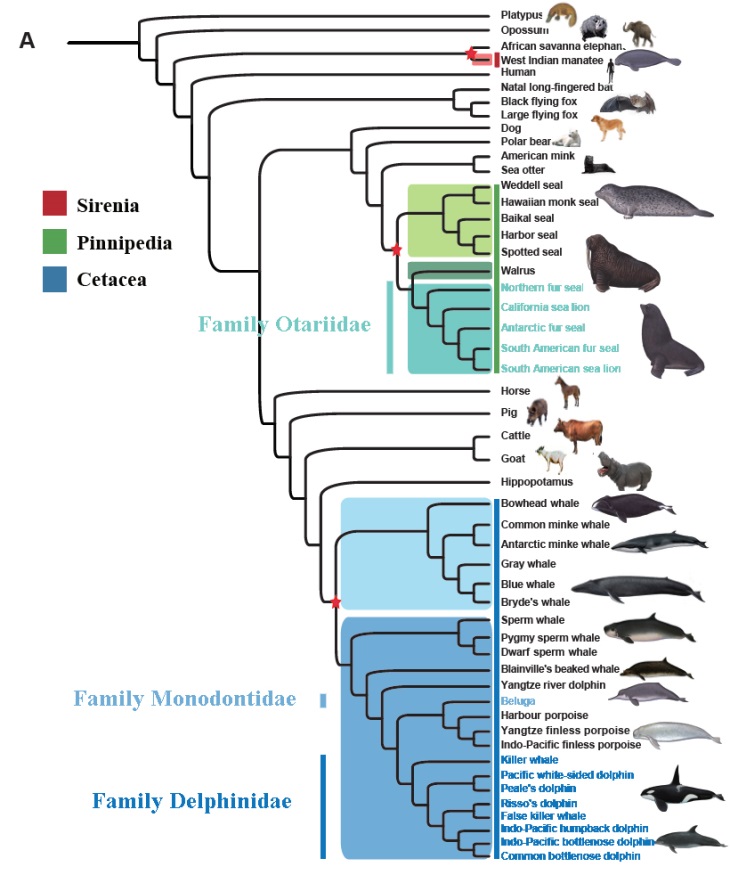
*

Figure S1. Phylogeny of marine mammals. Evolutionary position of the three investigated lineages (family Otariidae, Monodontidae and Delphinidae) are indicated in the chart.


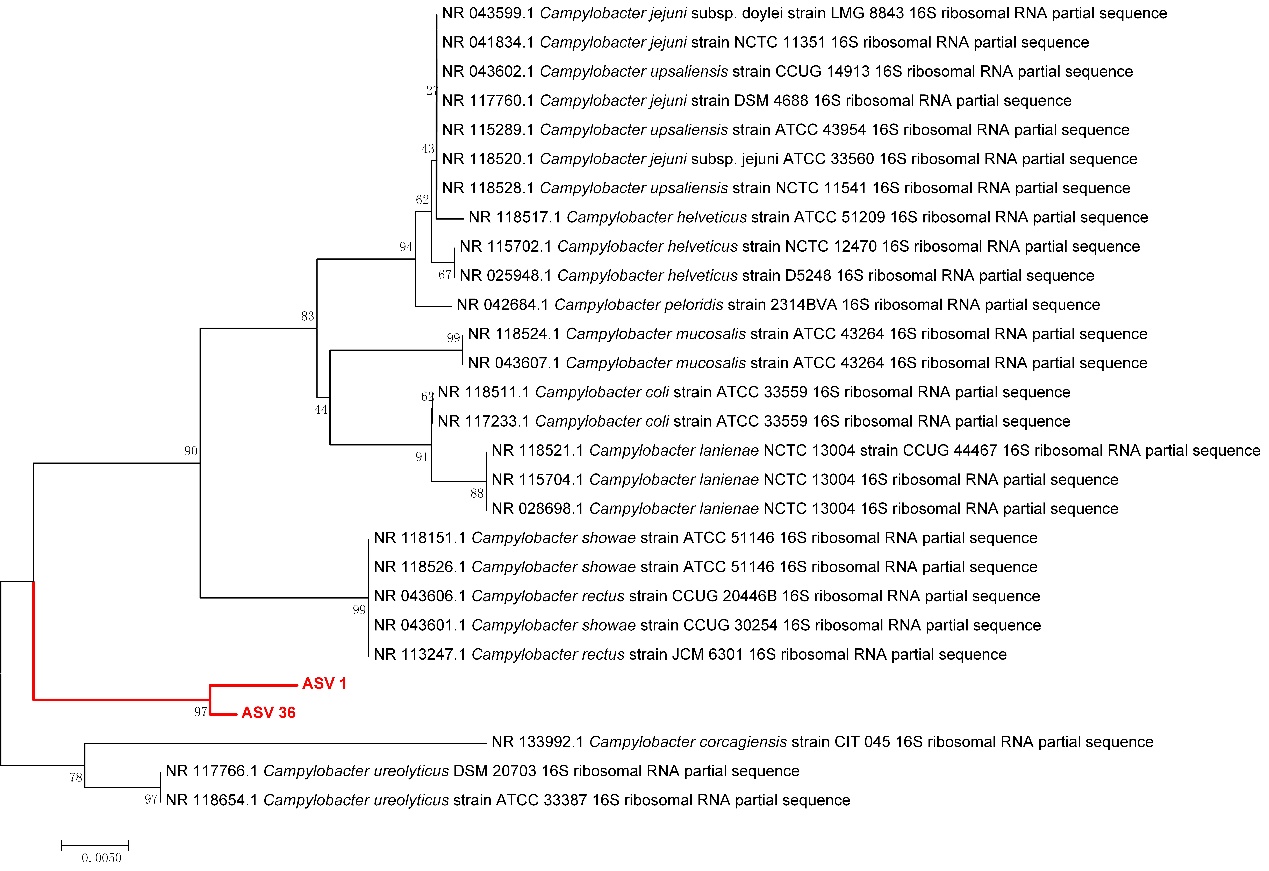


Figure S2. Phylogenetic tree based on the ASV1, and ASV36 sequences that show the relationships between each ASV and related strains by using the Neighbor Joining method with 1,000 replications.


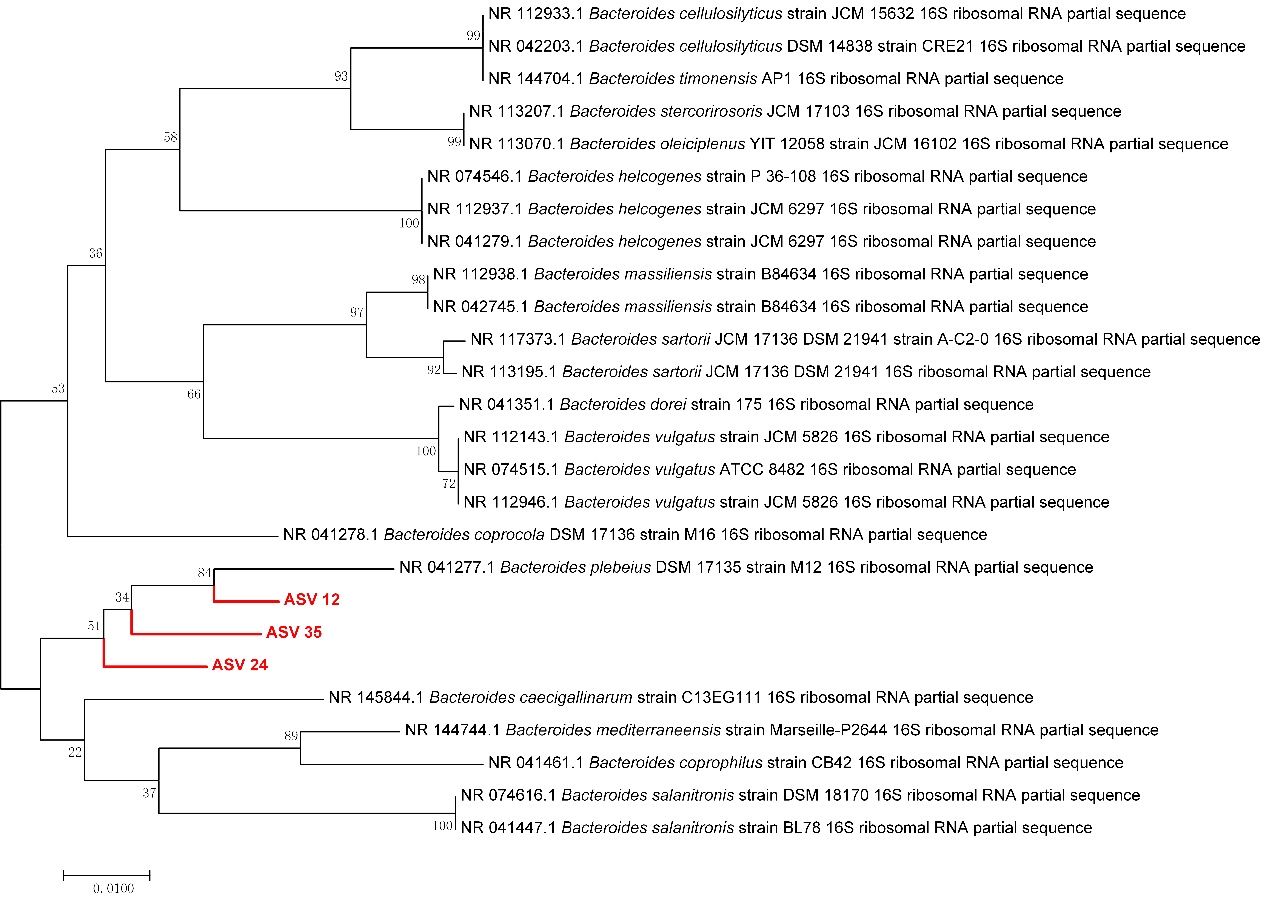


Figure S3. Phylogenetic tree based on the ASV12, ASV24, and ASV35 sequences that show the relationships between each ASV and related strains by using the Neighbor Joining method with 1,000 replications.


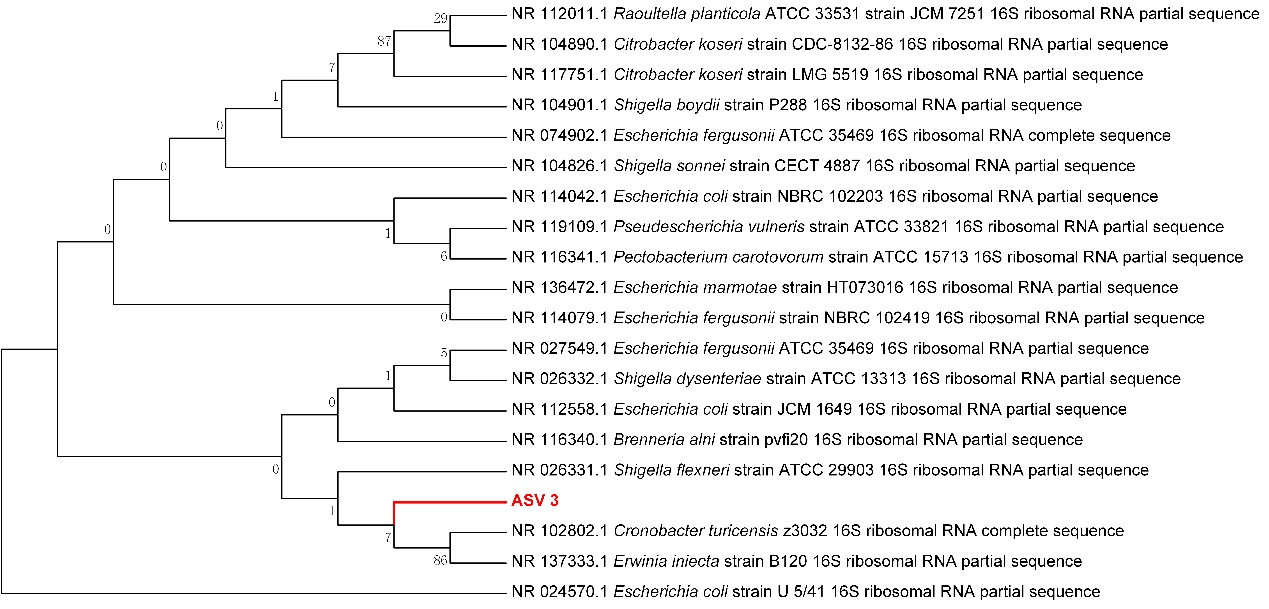


Figure S4. Phylogenetic tree based on the ASV3 sequence that show the relationships between ASV3 and related strains by using the Neighbor Joining method with 1,000 replications.


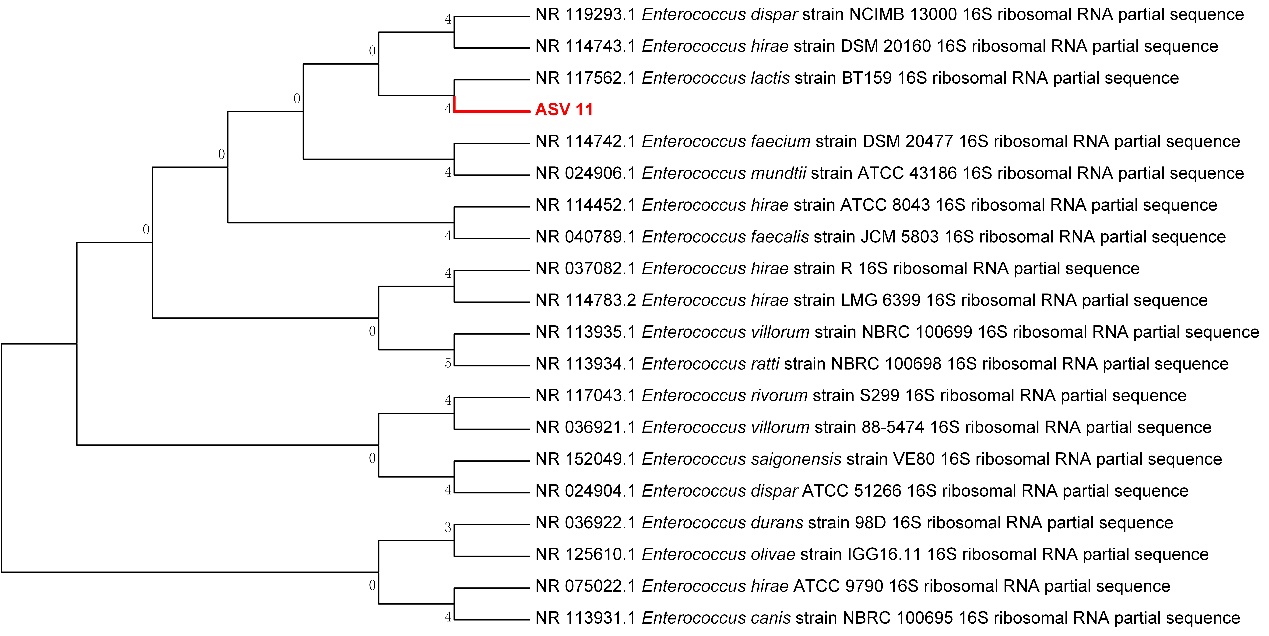


Figure S5. Phylogenetic tree based on the ASV11 sequence that show the relationships between ASV11 and related strains by using the Neighbor Joining method with 1,000 replications.


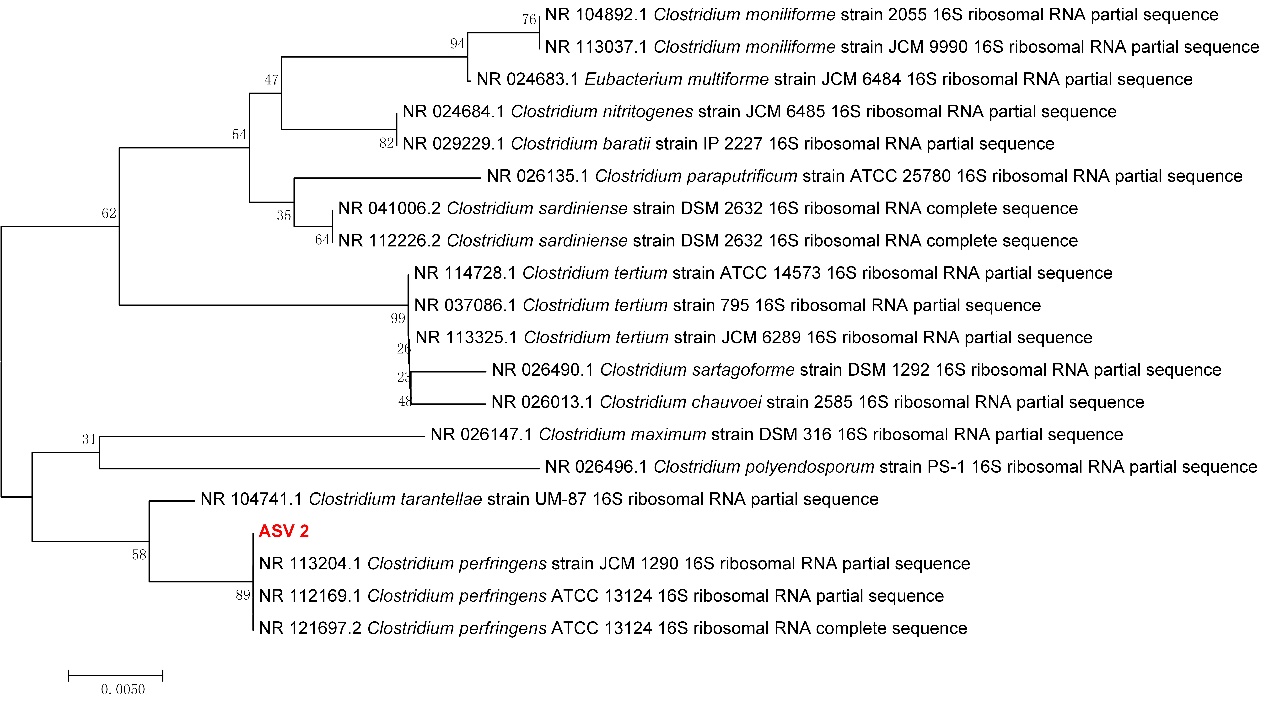


Figure S6. Phylogenetic tree based on the ASV2 sequence that show the relationships between ASV2 and related strains by using the Neighbor Joining method with 1,000 replications.


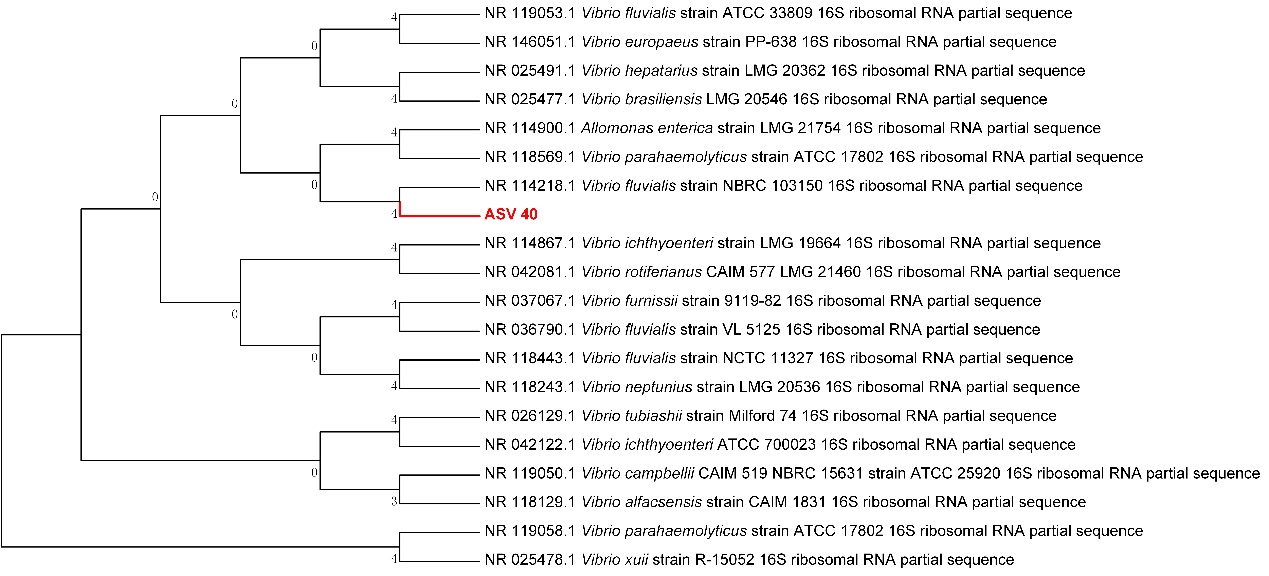


Figure S7. Phylogenetic tree based on the ASV40 sequence that show the relationships between ASV40 and related strains by using the Neighbor Joining method with 1,000 replications.


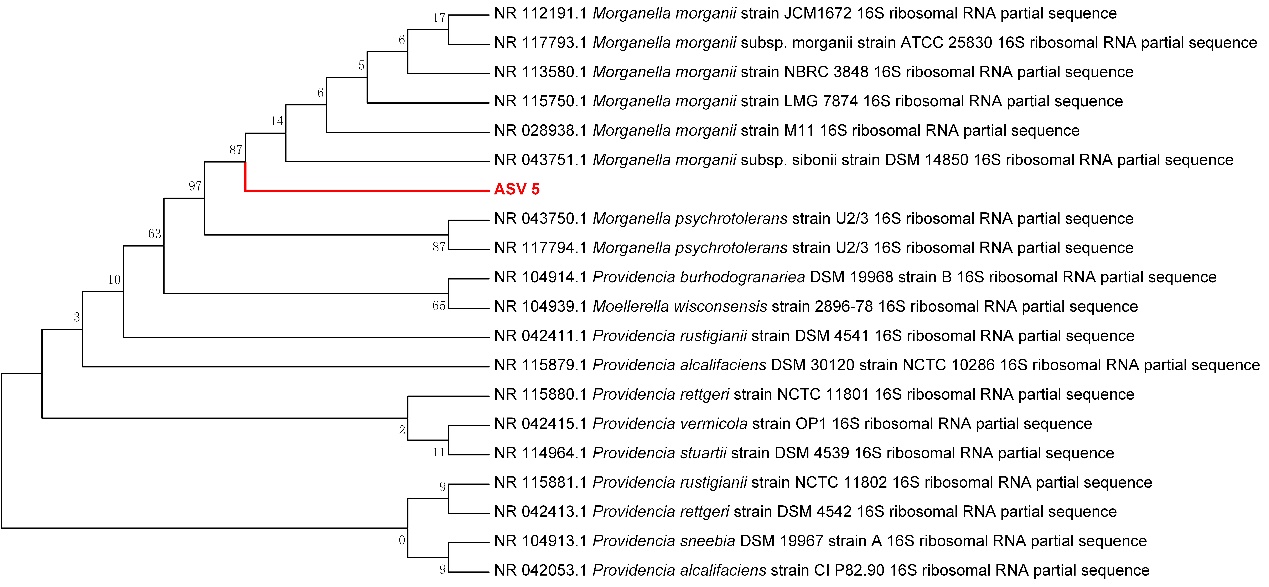


Figure S8. Phylogenetic tree based on the ASV5 sequence that show the relationships between ASV5 and related strains by using the Neighbor Joining method with 1,000 replications.
